# Quality of life and psychosocial aspects in Greek patients with psoriasis: a cross-sectional study*

**DOI:** 10.1590/abd1806-4841.20154147

**Published:** 2015

**Authors:** Anargyros Kouris, Christos Christodoulou, Christina Stefanaki, Miltiadis Livaditis, Revekka Tsatovidou, Constantinos Kouskoukis, Athanasios Petridis, George Kontochristopoulos

**Affiliations:** 1 "Andreas Sygros" Skin Hospital - Athens, Greece; 2 "Attikon" University Hospital - Athens, Greece; 3 University of Thrace, University Hospital of Alexandroupolis - Alexandroupoli, Greece

**Keywords:** Anxiety disorders, Depression, Psoriasis, Quality of life

## Abstract

**BACKGROUND:**

Psoriasis is a common, long-term skin disease associated with high levels of psychological distress and a considerable adverse impact on life. The effects of psoriasis, beyond skin affliction, are seldom recognized and often undertreated.

**OBJECTIVE:**

The aim of the study is to evaluate the quality of life, anxiety and depression, self-esteem and loneliness in patients with psoriasis.

**METHODS:**

Eighty-four patients with psoriasis were enrolled in the study. The quality of life, depression and anxiety, loneliness and self-esteem of the patient were assessed using the Dermatology Life Quality Index, Hospital Anxiety and Depression Scale, the UCLA loneliness Scale (UCLA-Version 3) and Rosenberg's Self-esteem Scale, respectively.

**RESULTS:**

The Dermatology Quality of Life Index score among psoriasis patients was 12.61 ± 4.88. They had statistically significantly higher scores according to the Hospital Anxiety and Depression Scale -anxiety subscale (p=0.032)-compared with healthy volunteers. Moreover, a statistically significant difference was found between the two groups concerning the UCLA-scale (p=0.033) and RSES-scale (p<0.0001). Female patients presented with lower self-esteem than male patients.

**CONCLUSION:**

Psoriasis is a distressing, recurrent disorder that significantly impairs quality of life. Therefore, the recognition and future management of psoriasis may require the involvement of multi-disciplinary teams to manage the physical, psychological and social aspects of the condition, as is the case for systemic, long-term conditions.

## INTRODUCTION

Psoriasis is a chronic, scaling inflammatory skin disease that affects approximately 1.5-3% of the population.^1^ Although it is not contagious or life-threatening, it can affect the patient's quality of life, with negative psychosocial implications. The burden of living with psoriasis is equivalent to or greater than that seen in other long-term conditions, such as cardiac failure and chronic lung disease, though it tends to be underestimated.^2^

The patient often feels stigmatized, which further intensifies their lack of self-confidence and self-esteem. Visible skin changes on the patient's body may cause aversion, attract negative attention, public rejection, reactions of disgust, and can provoke fear of contagion.^3^ On a social level, the stigma, feelings of shame, rejection, embarrassment and lack of confidence provoked by the disease, often lead to the discontinuation of daily activities and social withdrawal.^4^

Excessive alcohol consumption and increased smoking are common in patients with psoriasis, particularly those severely affected, compared with the general population. Poikolainen et al. reported an increased mortality ratio in the parameters of smoking- and alcohol-related causes in psoriatic patients.^5^ Alcohol and smoking can exacerbate the severity of psoriasis and impair treatment response.

Stressful life events constitute a major risk of occurrence and recurrence, aggravating the severity of the disease and duration of symptoms. Anxiety and depression can worsen the condition or cause resistance to treatment.^6^ In addition, the disease itself contributes to depression, anxiety and psychological stress, thus creating a "vicious circle" that is difficult to manage. Verhoeven et al. described how daily stressors trigger the severity of the disease and intensify itching, showing that patients with a higher tendency to worry are more vulnerable to the impact of stressors.^7^

In view of the high prevalence of psoriasis and the distress it causes, the object of the present study was to evaluate the quality of life, depression, anxiety, self-esteem and loneliness in patients suffering from this condition.

## MATERIALS AND METHODS

Patients with chronic plaque psoriasis attending the Outpatient Clinic at the Andreas Sygros Skin Hospital, Athens, Greece, from July 2012 to August 2013, were enrolled in the study. The diagnosis of psoriasis was based on clinical and histological grounds. They were evaluated at their first visit to the outpatient clinics by two independent investigators who had no participation or conflict of interest. They answered all questionnaires on their own and underwent all the evaluations.

The study protocol was approved by the Ethics Committee of the Andreas Sygros Skin Hospital and signed informed consent was obtained from all patients and healthy volunteers. Inclusion criteria were the following: aged over 18 years and ability to understand Greek. Individuals with previous psychiatric history and/or history of receiving psychotropic drugs were excluded from the study.

The severity of psoriasis in each patient was assessed by the Psoriasis Area and Severity Index (PASI-score).^8,9^ To assess patients' quality of life, the Dermatology Life Quality Index (DLQI) was applied.^10^ Evaluations were performed for the following: anxiety and depression, using the Hospital Anxiety and Depression Scale (HADS); self-esteem, applying Rosenberg's Self-esteem Scale (RSES); and loneliness, drawing on the UCLA loneliness Scale (UCLA-Version 3).^11-15^

The PASI score measures the clinical severity of the disease. Scores for erythema, desquamation and infiltration of the skin ranged from 1 to 4, and from 1 to 6 in the affected area. Thus the PASI ranges from 0 to 72, with higher scores indicating more severe disorders. PASI of under 3 received the score of 1 (mild), PASI > 3 and < 15 was graded as 2 (moderate), while PASI > 15 indicated a score of 3 (severe). The DLQI is a validated, 10-item questionnaire covering personal relationships, daily activities, leisure and treatment. The maximum score is 30, where 0 indicates the least impairment and 30 the greatest impairment to a patient's quality of life. The HADS is a self-report rating scale comprising 14 items, designed to measure anxiety and depression, the most likely factors to cause psychological distress in patients (7 items for each subscale). Responses to items are placed on a four-point Likert Scale of 0 to 3 (score range 0-21, for each subscale), where high scores indicate more symptoms. The HADS scale has been validated in the Greek population.^12^ In the general population, HADS values were estimated to be HADS: 9.1±6.1, HADS-A: 5.1±3.7 and HADS-D: 3.9±3.1.^12^ The RSES is a 10-item questionnaire answered on a four-point scale; its scores range from 0-30; scores of 15-25 are of normal range, while scores of below 15 suggest low self-esteem and scores of over 25 indicate higher than average self-esteem . The UCLA (Version 3) is a 20-item questionnaire which measures personal perceptions of loneliness and social isolation; higher UCLA scores indicate stronger feelings of loneliness and isolation. The UCLA scale has also been validated in the Greek population.^15^ In the general population, UCLA-loneliness scale values were estimated to be 40.08±9.50 for young people and 31.51±6.92 for the elderly.^14^

The control group included eighty-four healthy, age- and sex-matched volunteers from the general population (in our study, individuals from the hospital's administrative staff were randomly selected), with no personal or family history of psoriasis, psychiatric history, or history of receiving psychotropic drugs, who were recruited during the same time period. Individuals participated in the control group voluntarily, without receiving any financial compensation. They answered all the same questionnaires as patients, apart from the DLQI scale for which they gave their their own scores, and underwent all the evaluations.

### Statistical analysis

All data were analyzed using the statistical package for social science (SPSS 17.0) for Windows. Results were expressed as means +/-SD. The t-test was used for quantitative analyses and comparison of means; Pearson's coefficient correlation was also applied. The statistical significance was determined at the level of P value 0.05.

### RESULTS

In this cross-sectional study, eighty-four patients were enrolled, comprising forty-one females and forty-three males, aged 22-61 (48.40±14.06).

The control group included eighty-four individuals (forty males and forty-four females), aged 19-64 (mean 51.11±12.69). No statistically significant (NS) difference between patients and controls was documented as regards age (t=1,31, p=0.19). Comparisons of other demographic characteristics are shown in table 1.

**Table 1:** Association studies with Raynaud's phenomenon (RP) in 373 systemic lupus erythematosus patients

Characteristics	Patients	Controls
	n (%)	n (%)
**Age, years**		
Mean (SD), range	48.40 (14.06)	51.11(12.69)^1^
	22-6	19-64
**Gender**		
Male	43 (51.2%)	40(47.6%)^a^
Female	41 (48.8%)	44(52.3%)
**Marital status**		
Married	52 (62%)	51(60.7%)^b^
Single	32 (38%)	33(39.3%)
**Employment**		
Employed	49 (58.3%)	47(55.9%)^c^
Unemployed	35 (41.7	37(44.1%)
**Family history of psoriasis**		
Yes	31 (36.9%)	0(0%)^d^
No	53 (63.1 %)	84 (100%)

1 t-test, t=1.31, p>0.05, df=166, NS

a χ2=0.21, p>0.05 df=1, NS,

b χ2=0.87, p>0.05 df=1, NS

c χ2=0.09, p>0.05 df=1, NS

d χ2=74.53, p<0.001 df=1, (yate's correction)

The DLQI score among psoriasis patients was 12.61 ± 4.88. The results of the three questionnaires for both groups and the p-values of the quantitative comparisons between the two groups are presented in Table 2. Regarding the HADS-anxiety subscale, psoriatic patients had statistically significantly higher scores (9.64±4.31) compared with healthy controls (8.27±3.88) (p=0.032). In addition, a statistically significant difference was found between the two groups concerning the UCLA scale (p=0.033). As shown by the RSES scale, psoriatic patients had statistically significantly lower scores (14.75±2.95) compared with healthy controls (17.63±4.37) (p<0.0001), suggesting low self-esteem. Similarly, in accordance with the aforementioned cut-off scores from the Rosenberg scale, 51 patients scored under 15 while the remaining 33 scored over 15, versus 18 and 66 from the control group, respectively (x^2^=26.78, p<0.001, df=1).

**Table 2:** Anxiety, depression, self-esteem and social isolation in psoriasis patients and controls

	Psoriasis patients (n=84)	Controls (n=84)	p-value
Total score HADS-total mean ±SD	17.57±6.54	15.78±6.23	0.072
Total score HADS-anxiety mean ±SD	9.64±4.31	8.27±3.88	0.032
Total score HADS-depression mean ±SD	8.34±3.16	7.60±3.50	0.154
Total score UCLA mean ±SD	46.02±7.03	44.04±4.60	0.033
Total score Rosenberg mean ±SD	14.75±2.95	17.63±4.37	0.0001

t-test

No statistically significant difference was documented for the total HADS scale and the subscale HADS-depression (Table 2).The total score for the DLQI, UCLA, RSES and HADS-total scale, subscales HADS-anxiety and HADS-depression by gender, is displayed in table 3. A statistically significant difference was observed in the RSES scale (p=0.015).

**Table 3:** Anxiety, depression, self-esteem and social isolation across gender in psoriasis patients

	Psoriasis patients (n=84)	Controls (n=84)	p-value
Total score DLQI mean ±SD	12.44±4.31	12.80±5.46	0.736
Total score HADS-total mean ±SD	17.48±6.77	17.65±6.37	0.906
Total score HADS-anxiety mean ±SD	17.48±6.77	9.36±4.06	0.615
Total score HADS-depression mean ±SD	8.48±4.09	8.56±3.26	0.929
Total score UCLA mean ±SD	44.47±7.22	47.04±7.19	0.106
Total score Rosenberg mean ±SD	15.88±3.67	14.09±2.83	0.015

t-test

The mean Pasi score were 12.29±4.25 (range 3-23). Thirty-nine patients had PASI scores <15 and forty-five patients had PASI scores >15. The quality of life of patients with PASI scores >15 was significantly affected compared with patients whose PASI scores were <15 (p<0.0001). Moreover, patients with severe psoriasis had statistically significantly higher scores for the HADS-total scale, HADS-anxiety subscale and UCLA scale in relation to patients with moderate psoriasis (Table 4). A positive correlation was noted between the severity of the disease measured by PASI scores and DLQI (r= 0.571, p<0.0001), anxiety (r= 0.367, p=0.001), the total HADS scale (r= 0.250, p=0.022) and UCLA scale (r= 0.571, p<0.0001). In addition, a positive correlation was observed between the DLQI scale and HADS-depression subscale (r=0.244, p=0.025), HADS-anxiety subscale (r=0.221, p=0.044) and UCLA scale (r=0.529, p<0.0001).

**Table 4:** Anxiety, depression, self-esteem and social isolation in psoriasis patients with PASI scores of under 15

	Patients with PASI scores of <15 (n=39)	Patients with PASI scores of ≥15 (n=45)	p-value
Total score DLQI mean ±SD	9.35±3.93	16.17±2.83	0.0001
Total score HADS-total mean ±SD	14.89±6.57	18.04±5.76	0.022
Total score HADS-anxiety mean ±SD	7.83±4.43	11.17±4.14	0.001
Total score HADS-depression mean ±SD	7.82±4.41	9.13±2.83	0.104
Total score UCLA mean ±SD	41.29±6.54	49.57±5.52	0.0001
Total score Rosenberg mean ±SD	16.17±2.83	15.62±3.46	0.427

t-test

Nevertheless, no correlation was documented between age and DLQI, UCLA, RSES, anxiety, depression or total score for HAD-scale scores. Furthermore, no correlation was observed between UCLA-scale scores and RSES, anxiety, depression or total score for HAD-scale scores. Similarly, no correlation was noted between RSES-scale scores and UCLA-scale, DLQI, depression, anxiety or total score for HAD-scale scores.

## DISCUSSION

Psoriasis has a significant, negative impact on the patient's quality of life, representing a lifelong burden for them.^2,3^ Various environmental factors have been suggested as aggravating factors for psoriasis, including stress, physical trauma, excessive alcohol consumption and smoking.^1^ Rapp et al. reported that psoriasis can cause a reduction in physical and mental functioning comparable with that seen in arthritis, cancer, heart disease, hypertension, depression and diabetes. It also creates tremendous economic and financial burdens.^16^ The total annual cost for treating psoriasis is estimated to be between 1.6 billion and 4.3 billion dollars.^17^ Psoriasis has been associated with social isolation, low self-esteem, physical disability and psychosocial distress.^18^ The findings of this study confirm those of previous studies, as psoriatic patients in this study experienced significant impairment in quality of life, anxiety, low self-esteem, and social isolation. In addition, our results confirm that clinical severity is related to anxiety, social isolation and quality of life in psoriatic patients. This is consistent with previous studies showing that PASI scores have a positive correlation with DLQI scores.^19,20^ However, some studies mentioned weak correlations between quality of life and clinical severity.^21,22^ A possible explanation is that patients with worse health-related quality of life feel greater dissatisfaction with their current treatment compared with those who have a better health-related quality of life. Patients with psoriasis often experience social and psychological difficulties caused by their environment, problems with body image, self-esteem, feelings of stigma, and shame and embarrassment regarding their appearance.^23^ They may feel humiliated when exposing their bodies, e.g. during an intimate relationship, while swimming or using public showers, or living in conditions that do not offer adequate privacy.^24^ Many patients with psoriasis often feel the need to hide their condition, which severely affects their self-confidence.^25^ In the present study, patients also expressed feelings of low self-esteem which were correlated with gender but not with age or disease severity. Female patients presented with lower self-esteem than male patients, which could be explained by the fact that psoriasis causes visible skin changes, and as women seem to invest more in their personal appearance than men, this finding is unsurprising. Furthermore, no correlation was documented between age and quality of life, social isolation, anxiety or depression.

Psoriasis in itself causes stress, which further aggravates the condition. However, most of the patients who reported exacerbated episodes precipitated by stress described disease-related stress resulting from cosmetic disfigurement and the social stigma of psoriasis.^26^ Previous studies reported a depression prevalence rate ranging from 0 to 58% in psoriatic patients.^27^ One study showed that female patients appear to be more susceptible to depression than male patients. The incidence of anxiety is higher than that of depression among psoriatic patients. Moreover, patients have reported significantly higher degrees of anxiety than patients with other chronic disorders, such as cancer.^27^ In this study, patients had statistically significant higher scores according to the HADS-anxiety subscale compared with healthy controls, while no statistically significant difference was documented across gender.

As with other diseases, a trusting physician-patient relationship forms the foundation for effective therapy. Physicians should show understanding toward psoriatic patients because they are not only frustrated with the disease, but also with the level of care they usually receive or have received in the past. While sitting close to the patient at the beginning of the consultation, and palpating the lesions as part of the physical examination, physicians can assess how psoriasis has influenced the patient's life, and try to help them overcome inhibitions regarding social interactions. By establishing a trusting relationship, physicians will encourage patients to follow their recommendations concerning treatment and potentially ameliorate treatment compliance and outcomes.

## CONCLUSIONS

Psoriasis is a long-term skin disorder associated with significant impairment of the patient's quality of life and self-esteem, and high levels of distress which are frequently under-recognized. There is a need for pharmacologic interventions that should be accompanied by patient education and social and family support.
